# Genes Integral to the Reproductive Function of Male Reproductive Tissues Drive Heterogeneity in Evolutionary Rates in Japanese Quail

**DOI:** 10.1534/g3.117.300095

**Published:** 2017-11-20

**Authors:** Findley R. Finseth, Richard G. Harrison

**Affiliations:** * Keck Science Department, Claremont McKenna, Pitzer, and Scripps Colleges, Claremont, California 91711; † Department of Ecology and Evolutionary Biology, Cornell University, Ithaca, New York 14850

**Keywords:** reproductive protein, RNA-Seq, proteomics, comparative genomics, Japanese quail

## Abstract

Early comparative genomics studies originally uncovered a nonintuitive pattern; genes involved in reproduction appeared to evolve more rapidly than other classes of genes. Currently, the emerging consensus is that genes encoding reproductive proteins evolve under variable selective pressures, producing more heterogeneous divergence patterns than previously appreciated. Here, we investigate a facet of that heterogeneity and explore the factors that drive male reproductive tissue-based heterogeneity in evolutionary rates. In Japanese quail (*Coturnix japonica*), genes with enriched expression in the testes evolve much more rapidly than those enriched in the foam gland (FG), a novel gland that secretes an airy foam that males transfer to females during mating. We compared molecular evolutionary patterns among (1) genes with induced expression in breeding *vs.* wintering conditions for both tissues and (2) genes that encode foam proteins (FPs) *vs.* those with varying degrees of expression specificity in the FG. We report two major findings. First, genes upregulated in breeding condition testes evolve exceptionally rapidly, while those induced in breeding condition FGs evolve slowly. These differences hold even after correcting for hormonally-dependent gene expression and chromosomal location. Second, genes encoding FPs are extremely conserved in terms of gene identity and sequence. Together, these finding suggest that genes involved in the reproductive function of each tissue drive the marked rate of heterogeneity.

Reproduction is a fundamental property of all organisms and a key determinant of fitness. In sexually reproducing organisms, proteins manufactured from at least two individuals must perform a complex and intricate series of interactions to facilitate successful reproduction. Given this critical role in fitness, proteins involved in reproduction are intuitively viewed as conserved. Yet, the first studies emerging from evolutionary analyses of reproductive proteins showed them to be surprisingly diverse, and often among the most rapidly diverging genes in the genome (Swanson and Vacquier 2002; Nielsen *et al.* 2005; Clark *et al.* 2006; Turner and Hoekstra 2008a). Recently, however, studies suggest that reproductive proteins exhibit more heterogeneity in evolutionary rates than originally appreciated, with many proteins being under strong functional constraints and others revealing rapid rates of protein divergence (*e.g.*, Dean *et al.* 2009; Dorus *et al.* 2010; Finseth *et al.* 2014; Meslin *et al.* 2017).

An understanding of the myriad causes of heterogeneous evolutionary rates of reproductive proteins is beginning to emerge. The primary tissue of expression of a gene strongly impacts divergence, with testis genes often evolving very quickly due to the action of recurrent positive selection (*e.g.*, Dean *et al.* 2009; Grassa and Kulathinal 2011; Finseth *et al.* 2014). In addition to the particular tissue of expression, genes that are narrowly expressed generally evolve more rapidly than broadly expressed reproductive genes (Good and Nachman 2005; Dean *et al.* 2008; Grassa and Kulathinal 2011; Parsch and Ellegren 2013; Finseth *et al.* 2014). Chromosomal location of reproductive proteins (sex *vs.* autosome) can influence protein divergence through the Faster-X or Faster-Z effect (*e.g.*, Larson *et al.* 2016; Wright *et al.* 2015). Genes that are not constitutively expressed, which may be the case for many sex-limited photoperiod-sensitive reproductive phenotypes, are often under relaxed constraint (Van Dyken and Wade 2010; Meisel 2011). Additionally, a gene’s functional class (Dorus *et al.* 2006, 2010; Turner *et al.* 2008; Carnahan-Craig and Jensen-Seaman 2013; Good *et al.* 2013; Vicens *et al.* 2014), lineage specificity (Marshall *et al.* 2010; Grassa and Kulathinal 2011), degree of sex-bias (*e.g.*, Meisel 2011; Ellegren and Parsch 2007), essentiality (*e.g.*, Schumacher *et al.* 2017), and developmental timing (*e.g.*, Good and Nachman 2005; Larson *et al.* 2016) can all influence evolutionary rates of reproductive proteins.

Here, we seek to gain a better understanding of the mechanisms underlying heterogeneous evolutionary dynamics and focus on reproductive genes expressed in two tissues of male Japanese quail (*Coturnix japonica*): the testis and the FG (AKA the proctodeal gland). The testis and FG are both male-limited, photoperiod-sensitive, reproductive tissues that produce secretions passed to the female during reproduction (Coil and Wetherbee 1959; McFarland *et al.* 1968; Sachs 1969; Klemm *et al.* 1973; King 1981; Seiwert and Adkins-Regan 1998). While the testis produces sperm and seminal fluid, the FG generates copious amounts of an airy, meringue-like foam that males transfer to females during mating [Figure 1 LD (long day)]. Similar to many seminal fluids, foam increases fertilization efficiency (Adkins-Regan 1999; Sasanami *et al.* 2015), improves sperm motility, viability, and storage (Cheng *et al.* 1989b; Singh *et al.* 2011, 2012; Sasanami *et al.* 2015), and mediates the outcome of sperm competition (Cheng *et al.* 1989a; Adkins-Regan 1999; Finseth *et al.* 2013).

**Figure 1:**
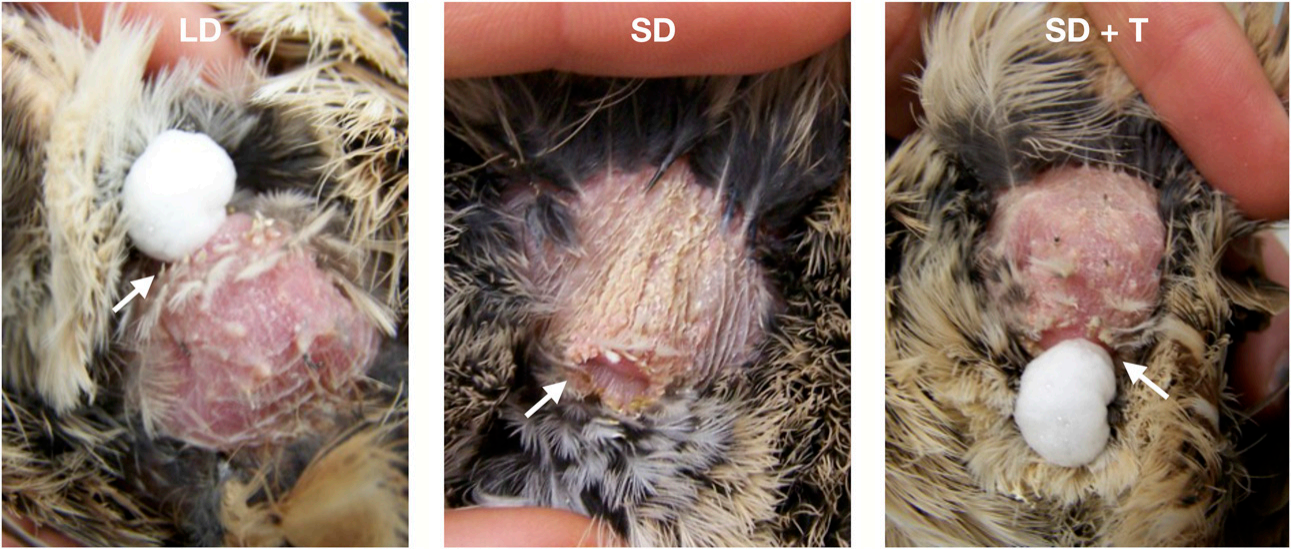
Photoperiod and hormonal manipulations cause the foam glands of male Japanese quail to regress and recrudesce. Photoperiod treatments mimicked breeding [*i.e.*, long day (LD)] or wintering [*i.e.*, short day (SD)] conditions. All males were implanted with either an empty implant or one filled with testosterone (T). All foam glands were gently squeezed prior to taking the picture. Treatments are as follows: LD, foam actively produced; SD, regressed foam gland and lack of foam production; and SD + T, foam gland recrudesced and foam actively produced. Pictures represent time point 3 (after hormone administration). Arrow indicates cloacal vent. Photos by F. Finseth and S. Iacovelli.

Male Japanese quail possess the unique FG and are the subject of our study. Both indirect and direct evidence suggests that sexual selection, and sperm competition in particular, is strong in Japanese quail. While the cryptic nature of Japanese quail has prevented direct characterizations of the mating system in the wild, studies of *C. japonica* from seminatural settings or of their sister species (*C. coturnix*) in the wild suggest a flexible mating system with opportunistic multiple mating (Nichols 1991; Teijeiro *et al.* 2003). Male Japanese quail also show phenotypes typical of species experiencing intense sperm competition, including large testes for their masses, a high daily output of sperm, and vigorous and forceful copulatory behavior (Clulow and Jones 1982; Møller 1991; Adkins-Regan 1995). Further, multiple inseminations are required to achieve natural levels of fertility and female Japanese quail can store sperm for up to 11 d, allowing ejaculates from different males to overlap, even when matings occur on different days (Sittmann and Abplanalp 1965; Birkhead and Fletcher 1994; Adkins-Regan 2015).

Previously, we characterized the selective pressures shaping genes with enriched expression in the FG and testis and discovered marked heterogeneity in evolutionary rates (Finseth *et al.* 2014). Repeated functional turnover in response to sexual selection is often cited as driving the pattern of relatively rapid divergence of many classes of reproductive proteins (Swanson and Vacquier 2002; Turner and Hoekstra 2008a; Wong 2011). Because both the FG and testis secrete proteins involved in sexual selection, we anticipated that genes deriving from either tissue would evolve rapidly due to a history of long-term, positive selection. Although genes with enriched expression in testes met our expectations, striking levels of selective constraint dominated the evolution of genes with enriched expression in the FG (Finseth *et al.* 2014). In fact, these patterns remained after correcting for expression levels, and increasing specificity of expression in the FG negatively correlated with evolutionary rate.

Here, we build on our previous study by probing the factors that resulted in the documented evolutionary rate heterogeneity and expand our analysis to examine how evolutionary origin and polymorphism map onto these factors. First, we explore how a combination of condition-dependent expression, chromosomal location, and primary tissue of expression influences evolutionary dynamics. This approach exploits the fact that the activity of both reproductive tissues changes seasonally and compares selective pressures shaping genes upregulated in breeding (*i.e.*, enlarged and active) *vs.* wintering (*i.e.*, regressed) conditions (Figure 1; categorizations in Figure 2A). Genes that are only expressed under certain conditions experience a relaxation of selective constraint (Van Dyken and Wade 2010), as do genes located on the Z chromosome [Wright *et al.* (2015), but see Sackton *et al.* (2014)]. Nonconstitutive expression and Z chromosomal location are predicted to increase evolutionary rate, but may not affect both tissues equally and could contribute to rate heterogeneity. This design also allows us to distinguish genes that encode the molecular/cellular “building blocks” of each tissue from the genes that are responsible for the reproductive role of each tissue. If the genes expressed by each tissue in its active state are under distinct selective forces, we may see differences in molecular evolutionary patterns even after correcting for conditional expression and chromosomal location.

**Figure 2:**
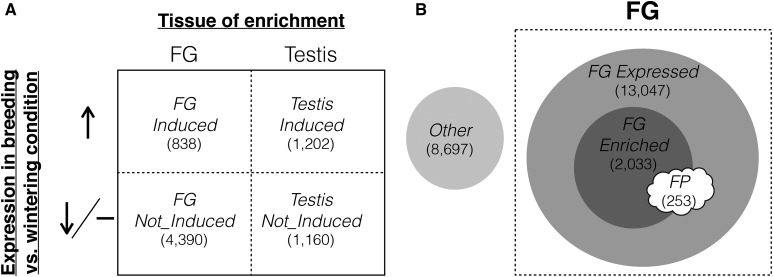
Genes were categorized into two functional groupings. (A) To identify genes integral to the sexual functions of foam glands (FGs) and testes, genes with enriched expression in FG or testes (Testis) were delineated as either significantly upregulated (Induced) or not (Not_Induced) in breeding (*i.e.*, enlarged and active) *vs.* wintering (*i.e.*, regressed and inactive) condition tissues. Enriched expression in a particular tissue relative to other tissues was determined in Finseth *et al.* (2014). (B) To compare genes that encode foam proteins (FPs) with those that vary for expression specificity in the FG, we used a combination of proteomics and RNA sequencing to identify genes that encode FPs, genes with enriched expression in the FG relative to two other tissues (FG Enriched), genes expressed in the FG (FG Expressed), and genes not expressed in the FG (Other). Each gene is represented in only one category determined by its least inclusive designation. Expression status in the FG was determined in Finseth *et al.* (2014). Sample sizes are given in parentheses.

Second, we investigate how gene function, tissue specificity, and chromosomal location influence the evolution of reproductive proteins. To this end, we characterize the protein constituents of foam, combining a mass spectrometry (MS) approach with RNA-Seq. The proteomic analysis allows us to characterize the genes that make the proteins that are transferred to females in the foam. Such transferred proteins could potentially interact with foreign molecules from pathogens, female reproductive tracts, or competing males, setting the stage for coevolutionary dynamics to produce a pattern of rapid evolution. We then explore whether the subset of genes expressed in the FG with the potential for coevolutionary interactions (*i.e.*, those encoding FPs) evolves in different ways compared to other genes expressed in the FG (as in Figure 2B). Again, we parse out the effect of chromosomal location on evolutionary rates.

## Materials and Methods

### Subjects

Unless stated otherwise, Japanese quail were lab-reared and housed on a 16L:8D light:dark cycle. Birds were housed individually at 4 wk of age (the onset of sexual maturity occurs at 6 wk). Males were prescreened for mating competency, and only those males who successfully mated with a female at least once were included in the study. Prior to the start of the experiment, all males were weighed, and their tarsus lengths and FG areas (length × width) were measured. Males were distributed randomly among treatment groups according to mass, mass/tarsus length (a proxy for condition), and FG area/mass. All animal procedures were approved by Cornell University’s Institutional Animal Care and Use Committee under permit 2002-0117.

### Experimental design and methods

Two groups of adult Japanese quail males were exposed to three treatments mimicking seasonal variation in FG and testis gene expression. Males in Group 1 were 12 months old at the start of the experiment (*N* = 6, experiments ran from November 2010 to February 2011). Males in Group 2 were 2 months old at the start of the experiment (*N* = 12, experiments ran from August to October 2011). Individuals from each group were distributed equally among three treatments—LD, short day (SD), and short day + testosterone (SD + T)—for a total of six males per treatment. LD males have functional FGs and testes that produce foam and sperm, SD males have regressed FGs and testes and do not make foam or sperm, and SD + T males have functional FGs and regressed, nonsperm-producing testes (Figure 1; Sachs 1967, 1969). Initially, all males were housed on long days (16L:8D light:dark cycle) to simulate breeding conditions. The SD and SD + T males were later placed on short days (8L:16D light:dark cycle) for either 7 (Group 1) or 3 (Group 2) wk prior to hormone implantation (below). The LD males continued on long days for the same amount of time.

All males were then surgically implanted with either empty (LD or SD) or testosterone-filled (SD + T; Sigma-Aldrich) implants according to their assigned treatment. Two Silastic implants (25 mm length, 1.6 mm inner diameter, and 2.4 mm outer diameter) of the appropriate treatment were placed subcutaneously in the neck/upper back region after numbing the skin with Bupivacaine (Sigma-Aldrich). The incision site was closed with 1–2 stitches and sealed with VetBond (3M). Implants were checked the following day for proper insertion and any remaining sutures were removed after 1 wk. Throughout the experiment, FG area, production, and volume (Group 2 only) were monitored on a weekly basis. We report measurements at three time points: (1) prior to photoperiod treatments (*i.e.*, baseline), (2) immediately preceding implantation (*i.e.*, after photoperiod treatment), and (3) 5 wk after implantation (*i.e.*, after hormone treatment).

Testosterone implants in SD males cause the FG to recrudesce and produce foam, but the testes remain regressed and do not produce sperm (Sachs 1969). Therefore, the SD + T treatment allowed us to control for gene expression differences in FGs that are determined by photoperiod, but not important for foam production. However, because SD + T males do not possess functional testes (*i.e.*, no recrudescence or sperm production), we were unable to have a similar control for testis-expressed genes. Prior to sample collection, we confirmed that all males exhibited the reproductive phenotypes appropriate to their treatment (LD: enlarged FG and testes, producing foam and sperm; SD: regressed FG and testes, not producing foam or sperm; and SD + T: enlarged FG but regressed testes, producing foam but not sperm) (Figure 1 and Supplemental Material, Table S1 in File S2). We killed subjects ∼5 wk after implantation and immediately dissected out the FGs from all males and testes from LD and SD males. Samples were immediately frozen in liquid nitrogen and later moved to −80° until RNA extraction.

### Library preparation and sequencing

We extracted RNA from 18 FGs (six males × three treatments) and 12 testes (six males × two treatments) with the Agencourt RNAdvance Tissue Kit (Beckman Coulter) following the manufacturer’s instructions, except that we used half-reactions. RNA quality and concentration were assessed by agarose gel electrophoresis and NanoDrop spectrophotometry. We confirmed RNA purity and integrity using an Agilent 2100 BioAnalyzer. In January 2012, we prepared 30 cDNA libraries from 1.2 μg total RNA using the TruSeq RNA Sample Preparation Kit (Illumina) following the manufacturer’s instructions. All samples were tagged with a unique adapter index, pooled, and single-end sequenced on three lanes of an Illumina HiSeq 2000, with a target read length of 100 bp. Sequencing was performed by the Cornell University Life Sciences Core Laboratories Center in April 2012.

Initial quality filtering and barcode removal were performed by the Genomics Facility at Cornell University’s Institute of Biotechnology. We used fastq-mcf (https://github.com/ExpressionAnalysis/ea-utils/blob/wiki/FastqMcf.md) to remove Illumina adaptors, trim low-quality terminal ends, discard short sequences, and filter reads with phred scores < 20. Trimmed reads from each FG sample were aligned to a published transcriptome made from liver, FG, and testis tissue (*N* = 81,868 transcripts; Finseth *et al.* 2014) using the aln algorithm of the Burrow–Wheeler transform in BWA version 0.6.2 (Li and Durbin 2009). The number of reads per sample uniquely mapped to each transcript was tabulated with samtools version 0.1.18 (Li *et al.* 2009). Similar approaches have yielded a high number of uniquely mapped reads appropriate for RNA-Seq in Japanese quail (Finseth and Harrison 2014).

### Characterization of genes upregulated in reproductively active FGs and testes

Previously, we characterized genes as exhibiting enriched expression in testes or FGs relative to other tissues [described in Finseth *et al.* (2014)]. From each set of tissue-enriched genes, we identified the subset that were significantly upregulated in the FGs or testes of LD males relative to expression in SD males using the multifactor glm approach in EdgeR version 3.2.3 (Robinson *et al.* 2010). Samples were normalized using the trimmed mean of M values approach (Robinson and Oshlack 2010). Negative binomial glms with Cox–Reid tagwise dispersion were fitted to models that included tissue, treatment (LD/SD), and male ID as factors. To filter out lowly expressed transcripts and reduce transcriptional noise, only transcripts with at least one aligned read per every million reads for at least six samples (*i.e.*, the number of biological replicates per treatment) were included. We removed any genes that were significantly upregulated in both tissues. Induced genes are those that are significantly upregulated in SD *vs.* either LD (FG, testes) or SD + T (FG only) males by more than log twofold change based on a false discovery rate of 5%. Any genes exhibiting significant enrichment in testes or FGs but not upregulated in reproductively active tissues were considered “Not_Induced” (*i.e.*, either downregulated or not differentially expressed; Figure 2A).

### Characterization of putative FPs using a combined RNA-Seq and proteomics approach

To identify transcripts that encode FPs, we combined RNA-Seq with a standard proteomics approach. First, RNA-Seq was used to detect genes significantly upregulated in FGs actively making foam. To this end, we tested for differential expression of transcripts using the multifactor glm approach in EdgeR version 3.2.3 (Robinson *et al.* 2010) as described above. Our design matrix specified contrasts to find genes differentially expressed in (1) LD *vs.* SD and (2) SD + T *vs.* SD. Transcripts that were (1) significantly upregulated in LD relative to SD, (2) significantly upregulated in SD + T relative to SD, and (3) represented by at least one aligned read per every million reads for at least six samples, comprised a list of candidate transcripts that are upregulated in reproductively active FGs and therefore may encode FPs (*N* = 2676).

We then compared this list of candidate genes to MS/MS data generated from the foam proteome to identify the protein constituents of foam. In brief, we pooled foam from six males, purified it, and ran the purified sample on a 1D SDS-PAGE gel for protein separation. Gel slices were digested with trypsin into peptides. The resulting peptides were extracted and fractionated using nano liquid chromatography prior to two rounds of MS (nanoLC-MS/MS). Spectra were searched against the predicted open reading frames of the *C. japonica* transcriptome. Matches at or above the 99% confidence threshold were considered confidently matched peptides. Proteins with at least two unique peptide matches comprised a preliminary list of 1006 genes encoding potential FPs (further details provided in supplementary methods, File S1). This was compared to the list of 2676 transcripts identified as significantly upregulated when foam is produced. The overlapping list of 253 transcripts we consider to be “high-confidence” FPs that are expressed in the FG. To assess how tissue specificity and gene function influence evolutionary rates, we compared this list of transcripts encoding foam FPs to genes with significantly enriched expression in the FG relative to testis and liver tissue (FG Enriched; *N* = 2038), transcripts expressed in the FG but also expressed in other tissues (FG Expressed; *N* = 13,047), and transcripts not expressed in the FG (Other = 8697). Categories were based on RNA-Seq data and are detailed in Finseth *et al.* (2014) (Figure 2B). Protein abundance, annotations, and GO term clustering analysis are described in the supplementary methods (File S1).

To validate our RNA-Seq data, we treated all FG samples of RNA with Turbo DNase (Ambion) and confirmed it to be free of genomic DNA. We then reverse-transcribed 200 ng of RNA into cDNA [SuperScript III, First Strand cDNA Synthesis Kit (Invitrogen)]. We designed primers from nine genes found to be upregulated in the FG of both SD *vs.* LD and SD *vs.* SD + T treatments (Table S2 in File S2). We verified that they amplified the intended target by Sanger sequencing and used β-actin as an internal control, as established previously (Finseth *et al.* 2014). Duplicate RT-qPCR reactions (25 μl) were conducted with the Power SYBR Green Master Mix (Applied Biosystems), starting with 33 ng of template and 200 nM of each primer. A ViiA seven (Applied Biosystems) thermocycler was used to perform reactions as follows: 95° for 10 min, 40 cycles of 95° for 15 sec, and 60° for 60 sec. We calculated primer efficiencies, ΔC_T_, and log fold change in the SD treatment *vs.* both of the two “foam active” treatments (SD + T or LD), as described by Zhao and Fernald (2005) (Table S2 in File S2). We tested for correlations between log fold changes generated by RT-qPCR and RNA-Seq for both treatments separately.

### Interspecific rates of protein evolution

We assigned quail:chicken orthologs using the reciprocal best BLAST method (Tatusov 1997; Bork and Koonin 1998; Koonin 2005). We compared the translated quail transcriptome to the chicken’s protein sequences (Ensembl version 69: *Gallus gallus* assembly WASHUC2) with a *e*-value cutoff of 1 × 10^−6^. Orthologs were called when the top hit (based on bit score) from the quail to chicken BLAST returned the original quail query in the chicken to quail BLAST (*N* = 9620 orthologs). Orthologs were assigned to either autosomal or Z chromosomes based on the WASHUC2 annotation. Chicken and translated quail protein sequences were aligned with Clustal W version 2.1 (Larkin *et al.* 2007). As implemented in the Parallel Alignment and Translation tool, version 1.0, PAL2NAL guided alignments of the corresponding DNA sequences (Suyama *et al.* 2006; Zhang *et al.* 2012). KaKs_Calculator was used to estimate pairwise evolutionary rates (*i.e.*, ω, the ratio of nonsynonymous (*d*_N_) to synonymous (*d*_S_) substitution rates; Zhang *et al.* 2006). We removed orthologs for which *d*_S_ > 2 times the mean *d*_S_ (as these might reflect poor alignment) and ortholog pairs for which *d*_S_ estimates approached 0, producing spurious ω values (ω ∼50; three ortholog pairs removed). We then examined whether the proportion of genes with orthologs and pairwise evolutionary rates varied according to (1) upregulation in breeding condition testes or foams glands (as in Figure 2A) and (2) specificity of expression in the FG (as in Figure 2B).

### Origin of genes

To identify the evolutionary origins of genes encoding characterized gene sets, we followed the general approach described by Knox and Baker (2008). First, we determined 1:1 single copy orthologs of transcripts with OrthoMCL (Chen *et al.* 2006). OrthoMCL combines a reciprocal best BLAST approach with a graph-clustering algorithm to identify homologous proteins and distinguish potential orthologs from paralogs. We restricted the list of confidently assigned orthologs to: (1) the single best hit based on BLAST similarity scores, (2) those where the best hit was from *G. gallus*, and (3) *C. japonica* transcripts represented by at least one aligned read per every million reads for at least six samples (*N* = 9774). Note that these criteria are slightly different than the one discussed in the *Interspecific rates of protein evolution* section, but identify similar numbers of orthologs (*N* = 9620). From this list, we identified the evolutionary origin of a quail transcript by finding the most distantly related species that possessed an orthologous gene (*i.e.*, had a member of the same orthologous group as identified by OrthoMCL). For each transcript, the most distantly related taxon with an ortholog was classed as Aves, Vertebrate, Animal, Eukaryote, or Bacteria + Archaea, based on the appropriate least inclusive group. For example, if the most distantly related species with an ortholog for a particular transcript was *Mus musculus*, that transcript would have been categorized as having a “Vertebrate” origin.

### Intraspecific polymorphism levels

RNA-Seq sequences from 12 male Japanese quail were used to compare intraspecific polymorphism levels among genes upregulated or not with enriched expression in the FGs or testes. This analysis relied on previously generated RNA-Seq data generated from livers, testes, and FGs of 12 males (Finseth *et al.* 2014). We merged bam files and removed duplicates using Picard Tools version 1.119 (http://broadinstitute.github.io/picard). The Unified Genotyper tool from the Genome Analysis Toolkit software suite version 2.8.1 was applied to perform SNP discovery (McKenna *et al.* 2010; DePristo *et al.* 2011). We calculated nucleotide diversity (π) in 500 bp windows with VCFtools version 0.1.12a (Danecek *et al.* 2011). Two separate ANOVAs were performed to determine significance. First, we assessed the contribution of upregulated expression in breeding condition testes and FGs on variation in average π values per gene (groupings as in Figure 2A). Second, we evaluated whether polymorphism levels varied according to the specificity of expression in the FG (groupings as in Figure 2B).

### Data analysis

Unless stated otherwise, all analyses were performed in R Version 3.0.1 (R Core Team 2014). C.I.s were generated in the Hmisc package of R, version 4.0-2 (Harrell 2014), by performing 10,000 bootstrap resamplings of the mean without assuming normality. Fisher’s exact tests and χ^2^ tests were used to test for significant differences among proportions. Unless stated otherwise, the false discovery rate was applied with a cutoff of 0.05 to call significance where necessary to correct for multiple testing (Benjamini and Hochberg 1995).

### Data availability

Raw data has been deposited at the National Center for Biotechnology Information’s Sequence Read Archive under BioProject ID PRJNA397592 and BioSample numbers SAMN07462512–SAMN07462553. Sample details are in Table S6 in File S2.

## Results

**Figure 3:**
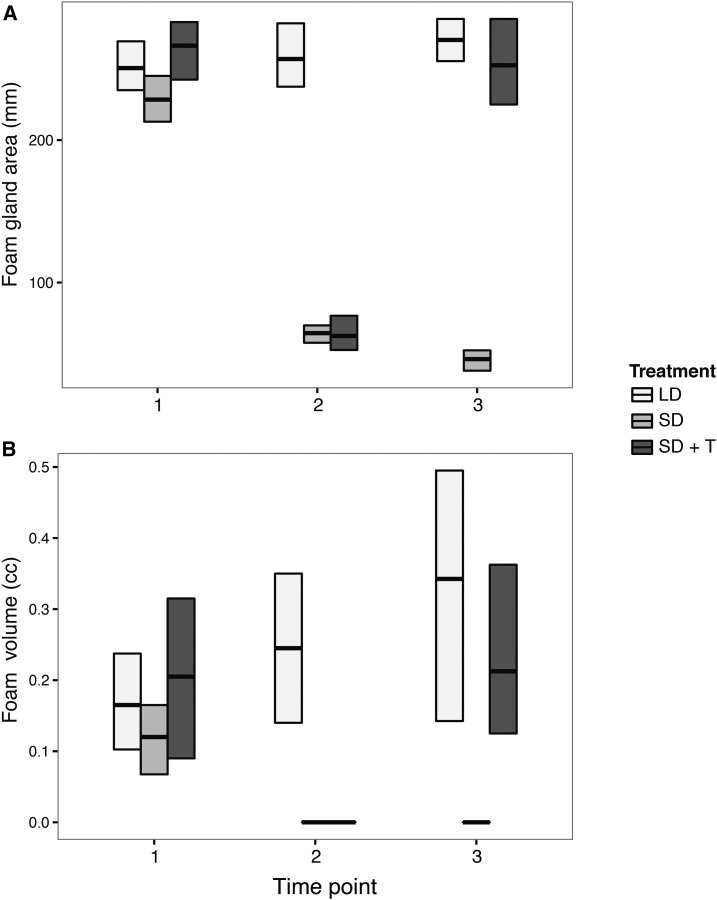
Both foam glands and foam production were affected by photoperiod and hormonal manipulations. Averages (bars) and 95% C.I.s (boxes) of (A) foam gland area and (B) volume for Japanese quail males at three different time points. Time points: 1 = baseline, 2 = after photoperiod treatment, and 3 = after hormone administration. Treatments: LD, long day; SD, short day; and SD + T, short day + testosterone. *N* = 6 (A) and *N* = 4 (B) per treatment as volume measurements were only taken for Group 2 (see *Materials and* *Methods*). C.I.s were derived from bootstrap resamplings of the mean without assuming normality.

Males responded to the light and hormone treatments as anticipated. The SD photoperiod treatment caused the SD and SD + T males’ FGs to regress and stop producing foam (Figure 3 and Table S1 in File S2). Implanting testosterone caused the FGs of SD + T males to recrudesce and produce normal volumes of foam (Figure 1, Figure 3, and Table S1 in File S2). Testes were regressed and did not produce sperm in both the SD and SD + T treatments (data not shown). The RNA-Seq and qPCR data were highly correlated for SD and either LD or SD + T treatments (SD−LD: *R*^2^ = 0. 9315, *F*_(1,7)_ = 95.16, and *P* = 2.519 × 10^−5^; and SD−SD + T: *R*^2^ = 0. 9624, *F*_(1,7)_ = 179.1, and *P* = 3.05 × 10^−6^).

### Functional groupings of transcripts

We sequenced a total of 30 samples: FGs from 18 males distributed across three treatments (LD, SD, and SD + T) and testes from 12 males distributed across two treatments (LD and SD) (Table S3 in File S2). Our first functional grouping distinguished genes that are upregulated in the breeding condition compared to the wintering condition (as in Figure 2A). For this grouping, we started with previously characterized genes that exhibited enriched expression in FGs (*N* = 2132) and testes [*N* = 5782; Figure 1 in Finseth *et al.* (2014)]. From these sets, we used RNA-Seq to identify the subset of genes that were upregulated in breeding condition FGs and testes (LD treatment) relative to regressed FGs and testes (SD treatment), excluding those that were upregulated in both tissues (FG Induced: 838 and Testis Induced: 4390). Genes from the tissue-enriched sets that that were downregulated or not differentially expressed in breeding condition tissues were considered “Not_Induced” (FG Not_Induced: 1202 and Testis Not_Induced: 1160). Our second functional grouping delineated genes according to their expression specificity in the FG, including whether or not they encode putative FPs (as in Figure 2B). We characterized 253 genes encoding putative FPs, as identified by a combination of RNA-Seq and mass spectrophotometry approaches (FP), 2033 genes with enriched expression in the FG relative to other tissues (FG Enriched), 13,047 genes expressed in the FG (FG Expressed), and 8697 genes not expressed in the FG (Other). For both functional groupings, we additionally characterized the subset of genes with 1:1 orthologs with chicken, and those orthologs that were alignable (grouping as in Figure 2A: FG Induced, 326; FG Not_Induced, 324; Testis Induced, 1462; and Testis Not_Induced, 239; grouping as in Figure 2B: FP, 210; FG Enriched, 636; FG Expressed, 5697; and Other, 1695). Details regarding protein identification, abundance, and enrichment are included in the supplementary results (Figure S1 and File S1).

### Genes induced in the breeding condition drive evolutionary rate heterogeneity

**Figure 4:**
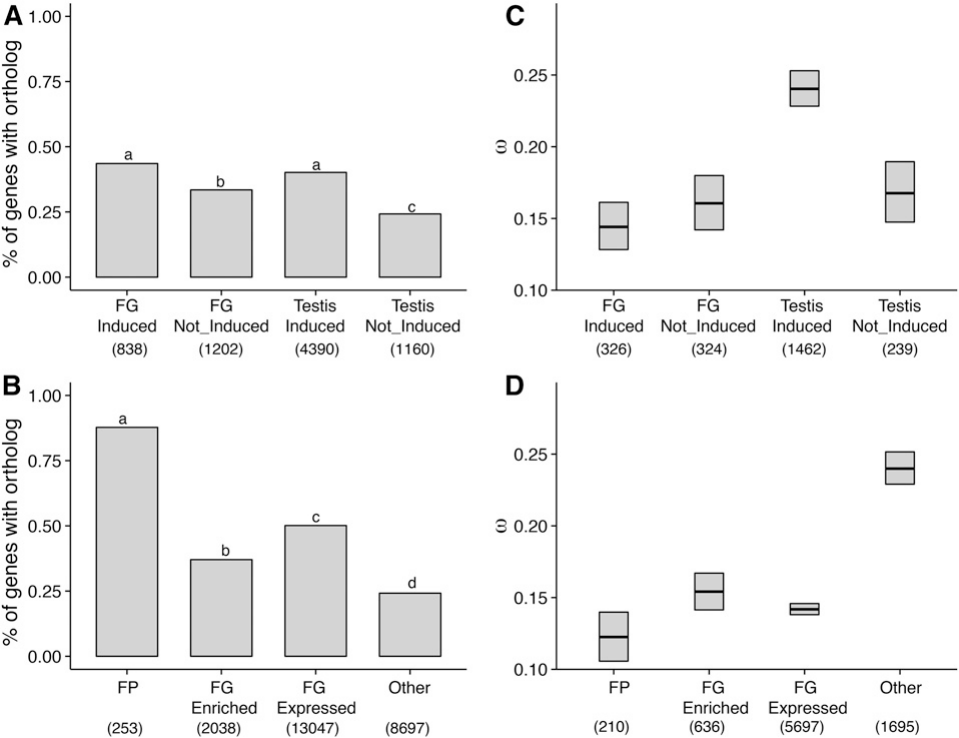
Genes induced in active testes evolve rapidly, but genes encoding foam proteins (FPs) are conserved in terms of orthology and rate of protein divergence. The proportion of genes with 1:1 orthologs in the chicken that are (A) induced during breeding vs. wintering condition FGs and testes and (B) expressed differentially in the FG. Mean pairwise ω estimates (bar) and 95% C.I.s (boxes) calculated from 1:1 orthologs between quail and chicken for genes (C) upregulated (Induced) or not (Not_Induced) in breeding *vs.* wintering condition foam glands (FGs) and testes, or (D) expressed differentially in the FG. C.I.s were derived from bootstrap resamplings of the mean without assuming normality. The percent of genes with 1:1 orthologs in the chicken that are (C) differentially expressed in breeding *vs.* wintering condition FGs and testes, and (D) expressed differentially in the FG. Bars with unique letters are significantly different based on pairwise Fisher’s exact tests (*P* < 0.05 after correcting for multiple comparisons). Samples sizes are given in parentheses and categorizations are described in Figure 2. Expression in the FG was determined in Finseth *et al.* (2014).

We previously reported pronounced heterogeneity in evolutionary rates from genes with enriched expression in testes and FGs; testis-expressed genes evolved rapidly, while FG-expressed genes evolved surprisingly slowly (Finseth *et al.* 2014). Here, we find that the heterogeneity remains after factoring in photoperiod-dependent expression and chromosomal location. When examining genes that are induced during the breeding condition, we see that testis genes still show markedly higher rates of evolution than FG genes (Figure 4A). Conversely, testis- and FG-enriched genes that do not increase in expression level when birds are in breeding condition (Not_Induced) show no difference in evolutionary rates. This raises two interesting points. First, genes that underlie the reproductive function of the testes (*i.e.*, those active in breeding condition), and not those that are the molecular/cellular building blocks of the tissues, are responsible for the previously observed differences in protein divergence. Second, genes that are not constitutively expressed are under relaxed selective constraint and would be predicted to evolve faster (Van Dyken and Wade 2010). As predicted, genes induced in testis breeding condition evolve faster than those not induced; there are no such patterns for FG genes (Figure 4A). An excess of *d*_N_ in testis-induced genes, rather than differences in *d*_S_, is responsible for the elevated evolutionary rates (Figure S2).

**Figure 5:**
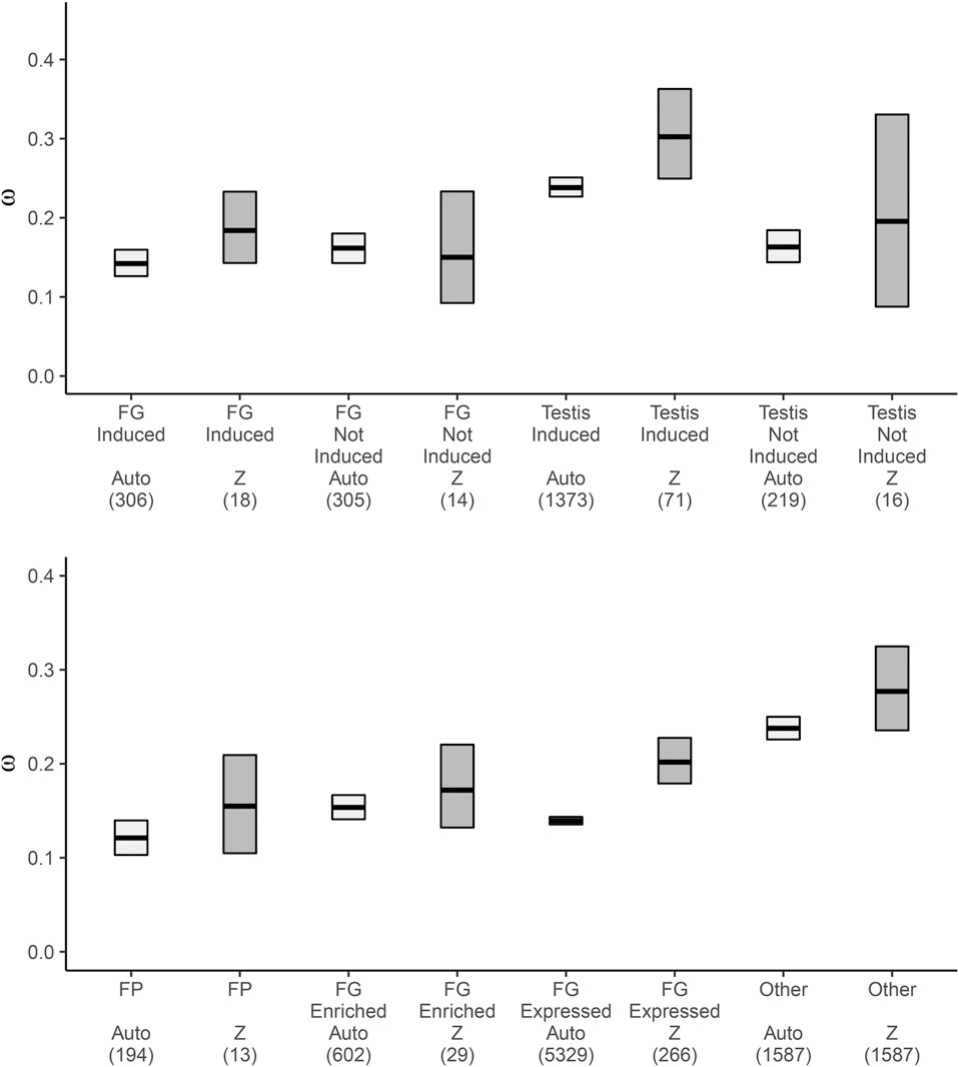
Faster Z-Effect does not drive evolutionary rate heterogeneity between two male-biased reproductive tissues. Mean pairwise ω estimates (bar) and 95% C.I.s (boxes) calculated from 1:1 orthologs between quail and chicken for autosomal (light gray) and Z-linked (dark gray) genes (top) upregulated (Induced) or not (Not Induced) in breeding *vs.* wintering condition FGs and testes, or (bottom) expressed differentially in the FG. Auto, autosomal; FG, foam gland; FP, foam protein; Z, Z-linked.

Importantly, after factoring in the chromosomal location of genes with chicken orthologs, we still see that induced genes drive rate heterogeneity across tissues. Testis-induced autosomal genes have significantly higher evolutionary rates than any other group of autosomally-derived genes (Figure 5). When considering genes on the Z chromosome, genes induced in breeding condition testes evolve faster than those in breeding condition FGs. For both Z and autosomal genes, evolutionary rates of genes not induced in the breeding condition are similar between tissues. Differences in the relative proportion of Z:autosomal genes from each tissue could also contribute to rate heterogeneity. However, we find that the frequency of genes found on the Z chromosome is similar across all tissues and treatments (Figure S3).

When considering the specificity of a gene’s expression in the FG, we similarly find that selective constraint is higher in genes that are more important to reproductive function (*i.e.*, that encode FPs; Figure 4B). Genes that encode FPs had slow rates of protein evolution (ω; 95% C.I.: 0.123–0.141); these ω values were significantly lower than values for genes specifically expressed in the FG (FG Enriched; 95% C.I.: 0.154–0.167) and nonspecifically expressed in the FG (FG Expressed; 95% C.I.: 0.142–0.146), and much lower than ω values for genes not expressed in the FG (Other; 95% C.I.: 0.240–0.250). Differences in *d*_N_, rather than in *d*_S_, drive this pattern (Figure S2). Genes that derive from autosomal or sex chromosomes at similar proportions in all categorizations and evolutionary rates of autosomally-derived FP genes are significantly lower than FG Enriched and Other genes, and trend lower than FG Expressed genes (Figure 5 and Figure S3).

### The foam proteome is comprised of highly conserved orthologs

Genes that evolve rapidly often have fewer orthologs identified in close relatives, as rapid sequence divergence can make orthology difficult to detect (*e.g.*, Bailey *et al.* 2013; Baker *et al.* 2012). We therefore hypothesized that genes involved in the reproductive function of the FG and testis, *i.e.*, induced during breeding condition or found in the foam proteome, would have fewer orthologs than other classes of genes. Instead, we found the opposite pattern. Genes upregulated in reproductively active FGs (FG Induced) or testes (Testis Induced) had similar proportions of orthologs in the chicken genome (0.43 and 0.40, respectively; *P* > 0.05), and these proportions were significantly higher values than for genes not upregulated in either tissue (FG Not_Induced: 0.33 and Testis Not_Induced: 0.24; *P* < 0.0001 for each comparison; Figure 4C). Moreover, genes that encode FP were disproportionately overrepresented by orthologs in the chicken genome (0.87) relative to those genes specifically expressed in the FG (0.37; FG Enriched), simply expressed in the FG (0.50; FG Expressed), or not expressed in the FG (0.24; Other; *P* < 0.00001 in all cases; Figure 4D).

**Figure 6:**
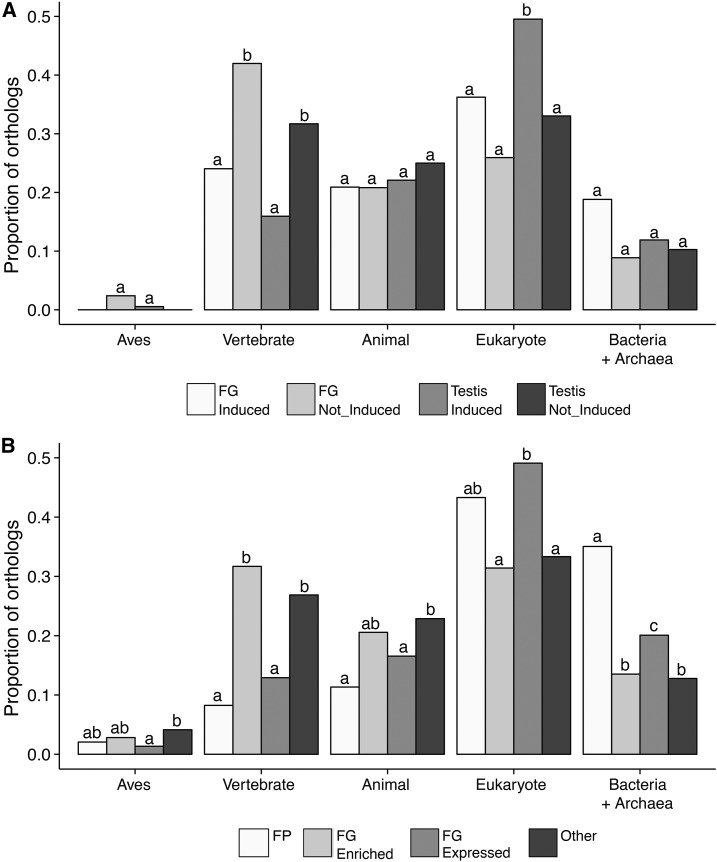
Genes induced in breeding condition male reproductive tissues arose in vertebrates, while foam proteins (FPs) are disproportionately represented by genes with orthologs in phylogenetically distant ancestors. Evolutionary origins of genes that are (A) induced or not in breeding condition foam glands (FGs) and testes, and (B) expressed differentially in the FG. The most distantly related species with an ortholog served as a proxy for the evolutionary origin of a gene. Analyses were restricted to those genes with 1:1 orthologs in chicken. Bars with unique letters are significantly different based on pairwise Fisher’s exact tests performed within each “origin” group. Sample sizes are as follows: FG Not_Induced, 293; FG Induced, 287; Testis Not_Induced, 224; Testis Induced, 1286; FP, 194; FG Enriched, 710; FG Expressed, 5782; and Other, 2151. Categorizations are described in Figure 2. Bars with unique letters are significantly different based on pairwise Fisher's exact tests (P < 0.05 after correcting for multiple comparisons).

To understand the evolutionary origin of genes that are critical to reproductive function, we identified the most phylogenetically distant orthologs for genes falling into our two functional groupings (Figure 2). When considering genes that are differentially regulated in breeding *vs.* wintering reproductive tissues (Figure 2A), we report two main results. First, for both FGs and testes, genes not induced at the onset of sexual activity (Not_Induced) were more likely to have a vertebrate origin than those upregulated during sexual activity (Figure 6A). This suggests that genes encoding the structural building blocks of each tissue were more likely to arise in vertebrates. Second, genes upregulated in testes were much more likely to have an older, eukaryotic origin than other classes of genes (Figure 6A). When considering relative expression in the foam proteome or FG (Figure 2B), the most striking result is that genes encoding FPs have the most ancient evolutionary origins (Figure 6B). Genes encoding putative FPs displayed a much higher proportion of orthologs found in Bacteria and/or Archaea than genes with enriched expression in the FG (FG Enriched), genes expressed in the FG (FG Expressed), or genes not expressed in the FG (Other).

### Intraspecific polymorphism levels are higher in genes upregulated during reproductive activity

Variable levels of selective constraint in the short-term can shape intraspecific polymorphism levels. We first examined intraspecific polymorphism levels for genes that are upregulated or not in reproductively active FGs and testes (Figure 2A). When considering nucleotide diversity levels (π), a gene’s expression status (upregulated or other), but not tissue of enriched expression (FG or testis), explained most of the variation. Genes that were upregulated in breeding conditions (mean π = 0.0027) revealed slightly, but significantly, higher levels of π than genes that were downregulated or not differentially expressed (mean π = 0.0024; ANOVA *F*_1_,_7144_ = 14.62, *P* = 0.00013). However, neither enriched expression in a particular tissue nor the interaction between tissue and expression status had a significant effect on average π levels per gene (tissue: ANOVA *F*_1_,_7144_ = 0.894, *P* = 0.344; and tissue × expression status: ANOVA *F*_1_,_7144_ = 0.415, *P* = 0.520).

We also examined how involvement in reproductive function influences selective constraint by exploring nucleotide diversity across genes with different expression specificity in the FG/foam proteome (Figure 2B). An ANOVA on nucleotide diversity values yielded significant variation in specificity categories (ANOVA *F*_3_,_22891_ = 16.313, *P* = 1.38 × 10^−10^). A *post hoc* Tukey test showed that nucleotide diversity levels did not differ between those genes that encode protein products present in the foam proteome and genes in any other category (FG Enriched, FG Expressed, and Other; *P* > 0.05 in all cases). However, genes that were simply expressed in the FG (FG Expressed; mean π = 0.0027) revealed slightly higher levels of polymorphism than genes with more specific expression in the FG (FG Enriched; mean π = 0.0025; *P* = 0.014) or not expressed in the FG (Other; mean π = 0.0025; *P* < 0.0001).

## Discussion

Genes encoding reproductive proteins often evolve at markedly different rates. We previously identified striking heterogeneity in the evolutionary rates of genes with enriched expression in two different reproductive tissues of male Japanese quail; those enriched in testes evolved quickly, while those enriched in the FG evolved under strong constraints (Finseth *et al.* 2014). To further investigate the underlying causes of this heterogeneity, we manipulated the reproductive state of male Japanese quail and examined the evolutionary dynamics of (1) the subset of genes that are upregulated in reproductive tissues when birds are in breeding condition (Figure 2A) and (2) genes that encode FPs (Figure 2B). We find that the genes responsible for the reproductive function of two male tissues drive heterogeneous evolutionary rates. Specifically, genes induced during testis recrudescence evolve rapidly at the sequence level, but genes integral to the sexual function of the FG are conserved in terms of both gene identity and sequence.

### Induced genes show conserved gene identity, but heterogeneous rates of divergence

Genes with enriched expression in the FG and testes were previously reported to exhibit different evolutionary rates; testis-enriched genes evolved rapidly and adaptively, but genes with enriched expression in the FG appeared to be under strong purifying selection (Finseth *et al.* 2014). To understand the processes that are responsible for these intriguing tissue-based differences, we used RNA-Seq to identify the subset of genes that are upregulated only when birds are in breeding condition, when each gland is enlarged and producing the secretions integral to their reproductive function (Figure 2A). We predicted that genes induced in the breeding state would show relatively rapid evolution for two reasons. First, they are more likely to be targets of sexual selection, often cited as producing rapid divergence of reproductive genes (Swanson and Vacquier 2002; Turner and Hoekstra 2008a; Wong 2011). Second, many are likely not constitutively expressed in these reproductive tissues and therefore may be under relaxed selective constraints (Van Dyken and Wade 2010).

Genes considered to be rapidly evolving often have few recognizable orthologs in closely related species due to high levels of sequence divergence or gene turnover (*e.g.*, Bailey *et al.* 2013; Baker *et al.* 2012). In contrast to our prediction, we found that genes induced in breeding condition tissues show proportionally more orthologs than those not upregulated, regardless of tissue of enrichment (Figure 4C). Additionally, for testis-upregulated genes, those orthologs tend to be of more ancient evolutionary origin (Figure 6A). However, when examining rates of protein divergence (ω), testis genes upregulated when birds are in breeding condition evolve at much faster rates than FG genes upregulated in breeding condition or testis genes that are not upregulated (Figure 4A). Taken together, the subset of genes induced in breeding condition reproductive tissues appears to be conserved in terms of gene identity, but still exhibits differences in rates of protein divergence across tissues. Importantly, this marked heterogeneity remained even after factoring in the effects of conditionally dependent expression and chromosomal location of genes (Figure 4A and Figure 5A).

### What drives heterogeneous evolutionary rates in reproductive proteins?

Why might genes upregulated in the breeding condition evolve so rapidly relative to FG-upregulated genes? Differential effects of both selective and neutral processes on each tissue may explain this pattern. First, testis-expressed genes may more often be targets of selection than FG-expressed genes. Indeed, we find that an excess of protein-changing substitutions in testis-induced genes are responsible for the divergent rates, suggesting a role for adaptive evolution acting on testis-biased genes (Figure S2). The proteins encoding the sperm itself are only found in testis-upregulated genes and could be partly responsible for this pattern. Many classes of sperm proteins, and in particular those that likely interact with foreign molecules, diverge quite rapidly in other species (Dorus *et al.* 2010; Vicens *et al.* 2014). The testes are also responsible for the production of seminal fluid, and many seminal fluid proteins in many nonavian species are the targets of natural selection [reviewed in Ram and Wolfner (2007)]. In birds, specific seminal fluid proteins are correlated with sperm performance and could be the targets of natural selection (Borziak *et al.* 2016). Consistent with greater adaptive evolution acting on testes, we previously identified numerous testis-enriched genes under positive selection and relatively large bursts of accelerated evolution along the quail lineage for testis-, but not FG-, enriched genes (Finseth *et al.* 2014).

Alternatively, testis-enriched genes may experience relaxed purifying selection relative to FG-enriched genes for numerous reasons. Coding sequences often evolve faster on sex *vs.* autosomal chromosomes, producing a Faster-Z or Faster-X effect. In avian ZW systems, neutral, nonadaptive processes appear to drive the Faster-Z effect due to increased genetic drift and a reduced efficacy of selection on Z chromosomes relative to autosomes [Wright *et al.* 2015; but see Sackton *et al.* (2014) for an adaptive explanation]. A disproportionate number of Z-derived genes in the testis could exhibit increased sequence divergence due to neutral processes, thereby producing the observed elevated evolutionary rates of genes with testis-enriched expression relative to those with enriched expression in the FG. Here, we tested that hypothesis by using chromosomal locations of chicken orthologs to dissect the Faster-Z effect. Overall, we found no evidence that rate heterogeneity is driven by the Faster-Z effect. When comparing among genes located on autosomes, we still find elevated evolutionary rates in testis-induced genes relative to all categorizations; similar trends remain when comparing among Z-derived genes (Figure 5). Further, there are no differences in the proportion of Z:autosomal genes in any tissue or treatment comparison (Figure S3).

Low expression level, narrow expression breadth, sex-limited expression, sex-biased expression, late developmental timing, and conditionally-dependent expression can also elevate protein divergence among species, without invoking natural selection (Meisel 2011; Van Dyken and Wade 2010; Wright *et al.* 2015; Dapper and Wade 2016). Previously, we showed that expression level and breadth do not drive the heterogeneity in evolutionary rate among reproductive tissues, as testis-enriched genes exhibited elevated divergence rates even after correcting for differences in expression level and tissue specificity (Finseth *et al.* 2014). In the current study, we explicitly manipulated conditions and found that heterogeneity remained among genes induced in the breeding condition (Figure 4A). Yet, we also found that a larger proportion of testis genes were induced in the breeding condition than FG genes, consistent with the hypothesis that there may be more relaxed constraint acting on the testes overall (Figure 2A). A related explanation is that the proportions of constitutively-expressed and sex-limited/sex-biased genes differ in the FG and testis. Our photoperiod manipulation would mitigate the effects of constitutive expression somewhat, but some of the genes that are induced in the breeding condition of each tissue may be expressed in other male (or female) tissues or at other developmental times. A comprehensive transcriptomic study of female and other male tissues across a developmental timelines could illuminate the role that these forces play (*e.g.*, Meisel 2011).

### FPs, which have the potential to interact with other gene products, are unusually conserved

Foam manufactured by the FG is transferred to the female reproductive tract during mating and influences the outcome of sperm competition (Finseth *et al.* 2014). Therefore, genes that encode FPs have the potential to coevolve with proteins from the female reproductive tract or from other males, and evolve under sexual selection. In this context, we expected that the subset of FG-expressed genes that encode FPs would also evolve relatively quickly. Contrary to the expectation of rapid evolution, we observe a striking degree of conservation among putative FPs as a class. Four lines of evidence allow us to draw this conclusion: (1) genes encoding FPs reveal significantly lower rates of protein evolution (ω) than genes encoding proteins not found in foam (Figure 4B), (2) FPs have more orthologs than other gene classes (Figure 4D), (3) these differences remain after accounting for chromosomal location (Figure 5 and Figure S3), and (4) these orthologs disproportionately trace back to phylogenetically distant groups (Figure 6B). Thus, the genes encoding FPs are partially responsible for the documented heterogeneity in evolutionary rates.

Why does the foam proteome evolve so slowly, despite involvement in sexual selection? Foam represents a novel proteome that is restricted to *Coturnix* quail (Klemm *et al.* 1973). Cooption of ancestral genes is a major theme in the evolution of novel phenotypes, with divergence in regulatory elements sometimes being more important than changes in protein sequence (True and Carroll 2002; Wray 2007; Carroll 2008). For example, genes with ancient evolutionary origins were coopted during the development of the placenta, likely due to modifications of regulatory elements (Knox and Baker 2008). More generally, the expression of male-derived reproductive genes evolves rapidly and may itself be a target of sexual selection (Nuzhdin *et al.* 2004; Khaitovich 2005; Ellegren and Parsch 2007). If foam functions mainly through emergent properties arising from new combinations/interactions of conserved proteins, diversifying selection on individual proteins may be weak. It is also possible that the aerated structure of foam, rather than individual chemical constituents, provides fertility benefits to males. The sperm of Japanese quail clump easily, and foam disaggregates sperm upon contact, which could be due simply to the structural matrix formed by foam (Singh *et al.* 2011). The bubbles in foam may also provide an oxygenated environment for sperm, thereby improving aerobic respiration. Similarly, selection resulting from sperm competition can target the rate of production of male reproductive proteins (Ramm and Stockley 2010), and the volume of foam transferred may be more critical than particular constituents.

Strong purifying selection on most of the foam proteome could also obscure adaptive evolution in a few important genes or codons that are responsible for the fertility benefits of foam. Pairwise estimations of evolutionary rates are inherently limited and may miss positive selection acting on one or a few codons. Additionally, rapid evolution can obscure orthology detection, and we only detected orthologs for 50% of FG-biased genes. While limitations of both pairwise evolutionary rate estimates and the detection of rapidly evolving orthologs could have caused us to miss rapidly evolving genes with FG-biased expression, these issues are equally or more problematic in genes with testis-enriched expression (Figure 4C). Therefore, our conclusion of relatively slow evolution of FG-biased genes when compared to testis-biased genes likely holds.

Intriguingly, the two most abundant proteins (by far) from our survey show signatures of rapid evolutionary dynamics (Table S4 in File S2). Specifically, Lysozyme, g-type 2, displays a relatively high pairwise evolutionary rate (ω = 0.4752), while *Coja17575_c0_seq* (which is expressed 22× more than any other gene) is either a novel gene or extremely divergent from its chicken counterpart; we were unable to identify an ortholog in chicken (Table S4 in File S2). In addition, we previously examined the subset of FG-enriched genes that are secreted and found that they evolve as rapidly as testis-secreted genes (Finseth *et al.* 2014). Taken together, this suggests contrasting dynamics between a few key FPs *vs.* the majority of FPs or genes expressed in the FG; abundant and/or secreted proteins may be targets of directional selection, whereas other FPs are exceptionally conserved.

### Genes upregulated during reproductive activity reveal higher levels of polymorphism

Patterns of polymorphism for male reproductive proteins, and seminal fluid proteins in particular, are complex. In some cases, accessory gland proteins show polymorphism patterns consistent with directional selection (*e.g.*, Begun and Lindfors 2005; Wagstaff and Begun 2005), including a reduction in intraspecific polymorphism as expected during a selective sweep (Kingan *et al.* 2003). However, more commonly, reproductive proteins reveal relatively high levels of polymorphism, which could be neutral or maintained by sexual conflict through balancing selection or negative frequency-dependent selection [*e.g.*, Tsaur *et al.* 2001; Metz and Palumbi 1996; Begun *et al.* 2000; Turner and Hoekstra 2008b; reviewed in Turner and Hoekstra (2008a)]. Given this context, we anticipated that the genes with the potential to be involved in sexual conflict (*i.e.*, those upregulated in active glands or encoding FPs) would reveal higher levels of polymorphism. While polymorphism patterns did not differ between genes encoding FPs and control panels of nonfoam genes (FG Enriched, FG Expressed, and Other), genes that are induced in breeding condition tissues were indeed significantly more polymorphic than those not upregulated in functioning tissues.

It is possible that the observed elevated intraspecific polymorphism levels in genes upregulated in breeding condition tissues are maintained through some form of balancing selection, but alternative explanations may be more likely. Polymorphism may be higher due to a relaxation of selective constraint on upregulated genes, particularly as these may be more narrowly expressed (Larracuente *et al.* 2008; Park and Choi 2010), conditionally-dependent (Van Dyken and Wade 2010), and less essential (Wolf *et al.* 2006) than genes that comprise the building blocks of the tissues (*i.e.*, are not upregulated in breeding condition). Further, the documented differences are minimal (difference in mean π = 0.0003), and it is unclear whether or not these slight differences are biologically meaningful.

Interestingly, despite differences in long-term divergence patterns between genes upregulated in FGs and testes (Figure 4C), we found no evidence for differences among tissues in levels of polymorphism. In *Drosophila*, some seminal fluid proteins experience episodic selection, with periods marked by strong directional selection alternating with periods dominated by neutral evolution or purifying selection (Begun and Lindfors 2005). Similar temporal variation in selection may be acting on the genes induced in breeding condition testes. Over the long-term, such cycles could produce major differences in selective signatures between such genes from the FG and testis (as revealed through the interspecific evolutionary rate comparisons) that are not captured by a single snapshot of polymorphism.

### Conclusions

The present study contributes to the growing appreciation for variable selective dynamics shaping the evolution of reproductive proteins and illuminates novel drivers of heterogeneous evolutionary rates. Specifically, we found that genes for which expression varies seasonally, with enlargement and regression of reproductive tissues, evolve under different selective regimes; moreover, these functional differences explain at least some of the tissue-based heterogeneity in evolutionary rates. Our approach is broadly applicable though not commonly used [but see Bogacka *et al.* (2017)], as many animals experience seasonal changes in the condition of reproductive glands. We also report contrasting patterns of conservation and divergence in FPs. The foam proteome includes many genes with evolutionarily ancient origins that are under selective constraint, but dominant FPs diverge rapidly. Additionally, for testes genes upregulated in the breeding condition, we document a difference between long- and short-term molecular evolutionary patterns that may be explained by alternating periods of adaptive and nonadaptive evolution. Taken together, simply being transferred to females and having the potential for coevolutionary interactions does not suffice for reproductive proteins to evolve rapidly.

## Supplementary Material

Supplemental material is available online at www.g3journal.org/lookup/suppl/doi:10.1534/g3.117.300095/-/DC1.
