# Supplementary material for: Genes Integral to the Reproductive Function of Male Reproductive Tissues Drive Heterogeneity in Evolutionary Rates in Japanese Quail
Source: G3 (Bethesda). 2017 Nov 20;8(1):39–51. doi: 10.1534/g3.117.300095 (PMC5765365; doi:10.1534/g3.117.300095)
Supplement: Supplementary file 1 [file 39FigureS1.pdf]

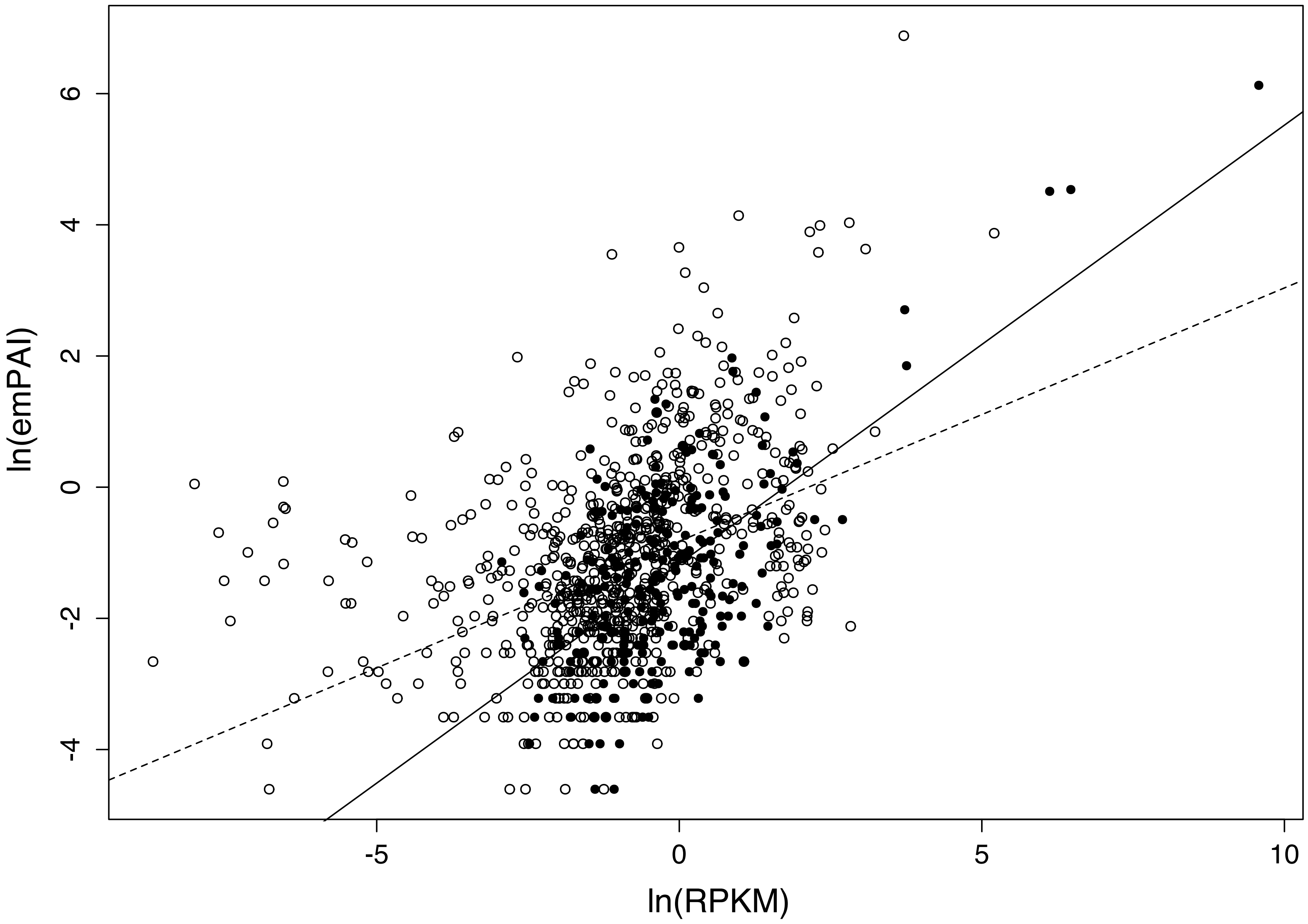

Figure S1. Protein abundance (emPAI) and expression level (RPKM) for genes that encode foam proteins are correlated. A significant relationship existed whether considering all genes that encode foam proteins identified proteomically (r^2= 0.1906, $F_{1,997}$ = 234.7, $P < 2.2 * 10^{-16}$; dashed line) and the subset of genes identified by both RNA-Seq and proteomics (*FP*; r^2 – 0.4209, $F_{1,251}$ = 169.4, P $< 2.2*10^{-16}$; solid line). Circles represent 999 foam genes identified proteomically and closed circles represent the 253 genes identified by both proteomic and RNA-Seq analyses (*FP*). Those genes with RPKM = 0 (N = 3) were removed from this dataset prior to analysis.
