# Supplementary material for: Genes Integral to the Reproductive Function of Male Reproductive Tissues Drive Heterogeneity in Evolutionary Rates in Japanese Quail
Source: G3 (Bethesda). 2017 Nov 20;8(1):39–51. doi: 10.1534/g3.117.300095 (PMC5765365; doi:10.1534/g3.117.300095)
Supplement: Supplementary file 2 [file 39FigureS2.pdf]

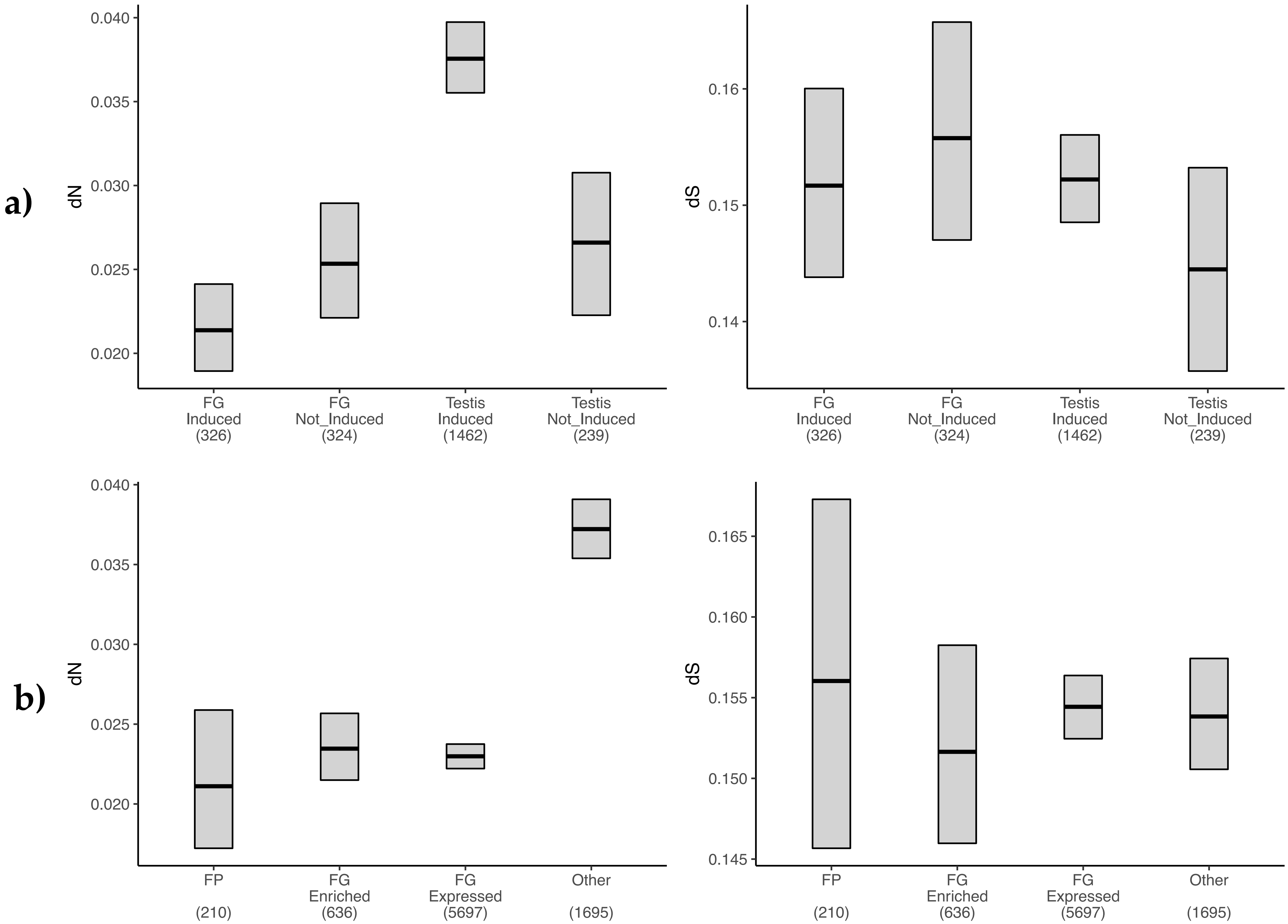

Figure S2. The rate of non-synonymous (dN) and synonymous (dS) substitutions of genes categorized according to **a)** induction in breeding state of either foam glands (FGs) or testes or **b)** specificity of expression in foam glands.
