# Supplementary material for: Genes Integral to the Reproductive Function of Male Reproductive Tissues Drive Heterogeneity in Evolutionary Rates in Japanese Quail
Source: G3 (Bethesda). 2017 Nov 20;8(1):39–51. doi: 10.1534/g3.117.300095 (PMC5765365; doi:10.1534/g3.117.300095)
Supplement: Supplementary file 3 [file 39FigureS3.pdf]

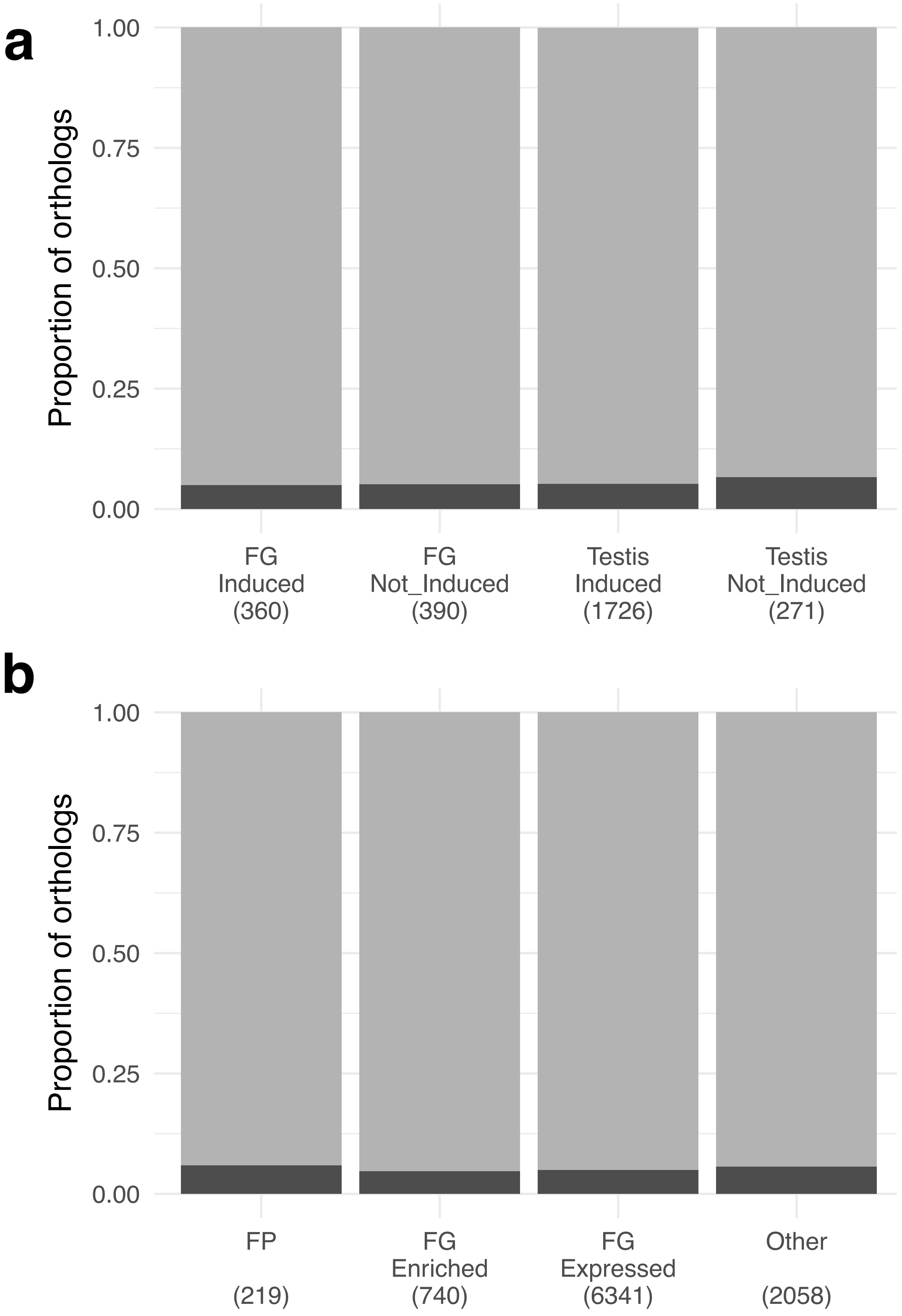

Figure S3. The proportion of genes located on the Z chromosomes does not differ across tissues and treatments. Quail transcripts with chicken orthologs were assigned to autosomal or Z chromosomes based on synteny to the chicken. Genes were grouped based on **(a)** whether or not they were induced in the breeding condition of foam glands and testes and **(b)** their specificity in the foam gland/proteome. Chi-square tests were performed to determine whether the Z:A proportions differed according to category (**a**: $X^2 = 1.0387$, df = 3, $P = 0.7919$; **b:** $X^2 = 2.0992$, df = 3, $P = 0.5521$). Groupings as in Figure 2.
