# Supplementary material for: Genes Integral to the Reproductive Function of Male Reproductive Tissues Drive Heterogeneity in Evolutionary Rates in Japanese Quail
Source: G3 (Bethesda). 2017 Nov 20;8(1):39–51. doi: 10.1534/g3.117.300095 (PMC5765365; doi:10.1534/g3.117.300095)
Supplement: Supplementary file 4 [file 39FileS1.docx]

**Supplementary text S1.**

**Supplementary methods.**

*Protein preparation and mass spectrometry*

Approximately equal volumes of foam were collected at 8 am from six sexually mature, 12-month old Japanese quail males and immediately prepared for SDS-PAGE protein separation. Each sample was suspended in 200 μL 1X phosphate buffered solution and centrifuged. The soluble fraction was transferred to a clean tube and left at room temperature for 4 hours to completely collapse the foam (air introduced during pipetting causes foam to reform). Equal parts of this solution from each male were pooled. We prepared, reduced, and denatured the pooled sample according to the NuPAGE® (Invitrogen™) instructions for denaturing gel electrophoresis under reducing conditions. Twenty μL of prepared sample were loaded onto a 4-12% NuPAGE® Novex Bis-Tris gel (Invitrogen™), separated with one-dimensional gel electrophoresis, and fixed according to the manufacturer’s instructions. The resulting 1D SDS PAGE gel was stained with Coomassie Blue solution (0.025% Coomassie, 10% acetic acid), de-stained overnight, and kept at 4 °C in deionized water until preparation for MS/MS two days later. Care was taken to avoid contamination of the gel with human proteins.

In-gel digestion, tryptic peptide extractions, and nanoLC-MS/MS were conducted by the Cornell University Institute of Biolotechnology Proteomics and Mass Spectrometry Facility. One lane from the 1D SDS PAGE prepared above was cut into 8 bands. Each band was reduced with 10mM Dithiothreitol (Roche), alkylated with 55mM Iodoacetamide (Acros Organics), digested with trypsin (Promega™ sequencing grade), then extracted twice with 50% acetonitrile (ACN), 5% Formic Acid (FA), and one final time with 90% ACN, 5% FA. The extracted peptides were lyophilized and reconstituted in 2% ACN, 0.5% FA for nanoLC-MS/MS analysis on an LTQ-Orbitrap Velos Mass Spectrometer (Thermo-Fisher Scientific, San Jose, CA) equipped with a CorConneX nano ion source (CorSolutions LLC, Ithaca, NY) and coupled to an UltiMate3000 nano Liquid Chromatograph (Dionex). Reconstituted peptides were injected onto a PepMap100 C18 trapping column (5µm particle size, 100Å pore size, 300µm inner diameter × 2cm length; Dionex) at 20μL/min for on-line desalting and separated on a PepMap100 C18 RP nano analytical column (3µm, 100Å, 75µm x 15cm; Dionex) installed on the CorConneX ion source with a 10µm emitter tip (NewObjective, Woburn, MA) and mounted in front of the instrument orifice. Peptides were eluted from the analytical column with a 60min gradient of 5 to 40% ACN in 0.1% formic acid at 300nL/min., followed by a 3min ramp to 95% ACN, 0.1%FA and a 5-min hold at 95% ACN, 0.1%FA. The column was then re-equilibrated with 2% ACN, 0.1%FA for 20mins prior to the next injection. The instrument was operated in positive polarity, with a nano spray voltage of 1.6 kV, an ion source capillary temperature of 275 °C, in data-dependent acquisition (DDA) mode using the FT mass analyzer for one survey (MS) scan of precursor ions followed by 10 MS/MS scans of the most intense ions with charge states +2 through +4 above a threshold ion count of 5,000 in the LTQ mass analyzer. MS survey scans were acquired at a resolution of 60,000 (fwhm at *m*/*z* 400) for the mass range of m/z 300-1800 and dynamic exclusion parameters were set at repeat count 1 with a 30s repeat duration, exclusion list size of 500, 15s exclusion duration, and ±10 *ppm* exclusion mass width. Collision induced dissociation (CID) parameters were set at the following values: isolation width 2.0 m/z, normalized collision energy 35 %, activation Q at 0.25, and an activation time of 10 ms. Internal calibration using the background ion signal for polysiloxane at m/z 445.120025 as a lock mass in addition to external calibration of the FT mass analyzer was performed. All data were acquired using Xcalibur 2.1 software (Thermo-Fisher Scientific, San Jose, CA).

All MS and MS/MS spectra were processed using Proteome Discoverer 1.3 (Thermo-Fisher Scientific, San Jose, CA), and the raw data were exported as MGF files for subsequent database search using Mascot Daemon (version 2.3.02, Matrix Science, London, UK). The open reading frames were predicted for all transcripts from a previously generated transcriptome (described in Finseth et al., 2014; N = 81,868) with OrfPredictor (Min et al., 2005). The acquired spectra were searched against these protein sequences with one missed cleavage by trypsin allowed. Peptide mass tolerance was set to 20 ppm and MS/MS mass tolerance was set to 0.8 Da. Carbamidomethylation of cysteine was set as a fixed modification, oxidation of methionine as well as deamidation of asparagine and glutamine were set as variable modifications. All matches at or above the 99% confidence threshold were considered confidently matched peptides. Proteins with at least 2 unique peptide matches, at least one of which matched only a single region in the transcriptome, constituted a preliminary list of 1006 potential genes encoding foam proteins.

*Protein abundance and annotations*

Protein abundance was calculated with the exponentially modified protein abundance index (emPAI) statistic (Ishihama *et al.*, 2005). For each transcript, expression levels were estimated as reads per kilobase per million mapped reads (RPKM; Mortazavi *et al.*, 2008). To evaluate whether protein and expression levels were correlated, we regressed emPAI on average RPKMs when foam was active (LD and SD + T treatment groups). The data were log-transformed prior to regression (excluding proteins with RPKMS = 0).

We annotated quail transcripts by identifying orthologous chicken sequences using the reciprocal best BLAST approach described in the Materials and Methods. We chose to annotate the entire proteomic dataset (N = 1006), rather than the list of high confidence proteins that overlap the RNA-Seq data (*FP;* N=253), because we noticed that several abundant proteins were not found in the RNA-Seq dataset. This suggested that these proteins are found in the foam proteome, but not manufactured in the foam gland; they are therefore not relevant for our questions about causes of variable heterogeneous evolutionary rates across tissues. However, because we were also interested in potential functions of foam proteins, we chose to annotate them.

We removed common contaminants from the protein set (*e.g.*, keratin) unless they were also identified in the RNA-Seq dataset for a total of 1002 foam proteins. The resultant list was annotated with respect to eight criteria: 1) identification in the protein and/or gene expression analyses, 2) fold change in foam inactive versus active glands, 3) rate of protein evolution, 4) enriched gene expression in a tissue (described in Finseth et al., 2014), 5) the ortholog hit ratio (*i.e.*, the amino acid sequence length of the quail protein, standardized by the length of its chicken ortholog; O'Neil *et al.*, 2010; Van Belleghem *et al.*, 2012), 6) the presence of a signal peptide, 7) Gene Ontology (GO) biological process and molecular function terms, and 8) protein classes. We predicted the presence of a signal sequence in SignalP 4.1 using default parameters (Petersen *et al.*, 2011). GO terms and protein classes were obtained from the PANTHER database (<http://www.pantherdb.org>).

GO biological process and molecular function terms were clustered according to similarity using the DAVID tool version 6.7 (Huang *et al.*, 2008; 2009). Using a modified Fisher’s exact test (*i.e.*, EASE score; Huang *et al.*, 2008), we tested for enrichment of clusters of terms in the genes encoding foam proteins (N = 1002 genes identified from proteomics analyses) compared with expectations from the filtered transcriptome from Finseth et al. (2014). Enrichment scores above 1.3 were considered significant, because 1.3 is equivalent to a non-log scale value of 0.05 (Huang *et al.*, 2008).

*qPCR validation of RNA-Seq data*

**Supplementary results.**

*Protein identification, abundance, and enriched GO terms*

Protein abundance (emPAI) and gene expression level (RPKM) were correlated for genes that putatively encode foam proteins (Figure S1). The correlation is stronger for the subset of genes identified by both proteomics and differential gene expression analyses (*FP*; r^2^ = 0.4209, F_1,251_ = 169.4, *P* < 2.2 * 10^-16^), than for those identified only proteomically (r^2^ = 0.1906, F_1, 997_ = 234.7, *P* < 2.2 * 10^-16^). We report the annotated dataset of 1002 proteins according to emPAI in Table S4. We clustered annotations of biological processes and molecular functions according to term similarity and evaluated whether certain clusters were enriched in the dataset of 1002 proteins compared to transcriptome expectations. This dataset is highly enriched for biological processes involving glycolysis and carbohydrate metabolism (Table S5: Annotation clusters 1 and 2), and significantly enriched (*i.e.,* enrichments score > 1.3) for biological processes associated with the regulation of actin cytoskeleton organization and filaments, protein glycosylation of the N terminus, and protein localization (Table S5). Genes encoding foam proteins were significantly enriched for molecular functions encompassing GTP binding, peptidase inhibitor activity, nucleotide binding, and intramolecular oxidoreductase activity (Table S5).
